# Supplementary material for: Proof-of-Concept Human Organ-on-Chip Study: First Step of Platform to Assess Neuro-Immunological Communication Involved in Inflammatory Bowel Diseases
Source: Int J Mol Sci. 2023 Jun 24;24(13):10568. doi: 10.3390/ijms241310568 (PMC10341384; doi:10.3390/ijms241310568)
Supplement: Supplementary file 1 [file ijms-24-10568-s001.zip › ijms-2444716-supplementary.pdf]

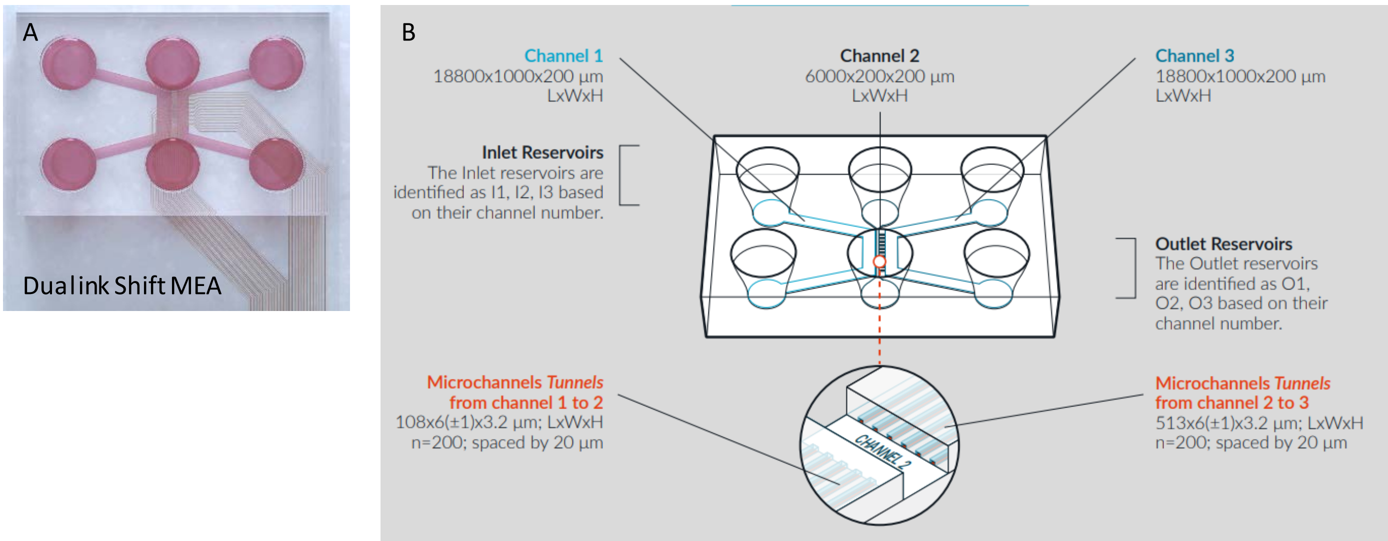

Figure S1: Asymmetric microfluidic device, called Dualink Shift. A: Illustrative photography of the asymmetric microfluidic device, called Dualink Shift MEA, composed of three compartments (pink). By transparency, it appears the MicroElectrode Array (MEA). B: Technical schema for specification of the asymmetric microfluidic device. Detailed presentation of the lengths (L), widths (W), and heights (H) of the microchannels (from channels 1 to 2 and 2 to 3) and three channels (channels 1, 2, 3). The dimensions of channels 1 and 3 (where the glutamatergic neurons are seeded) are 18800x1000x200 µm.

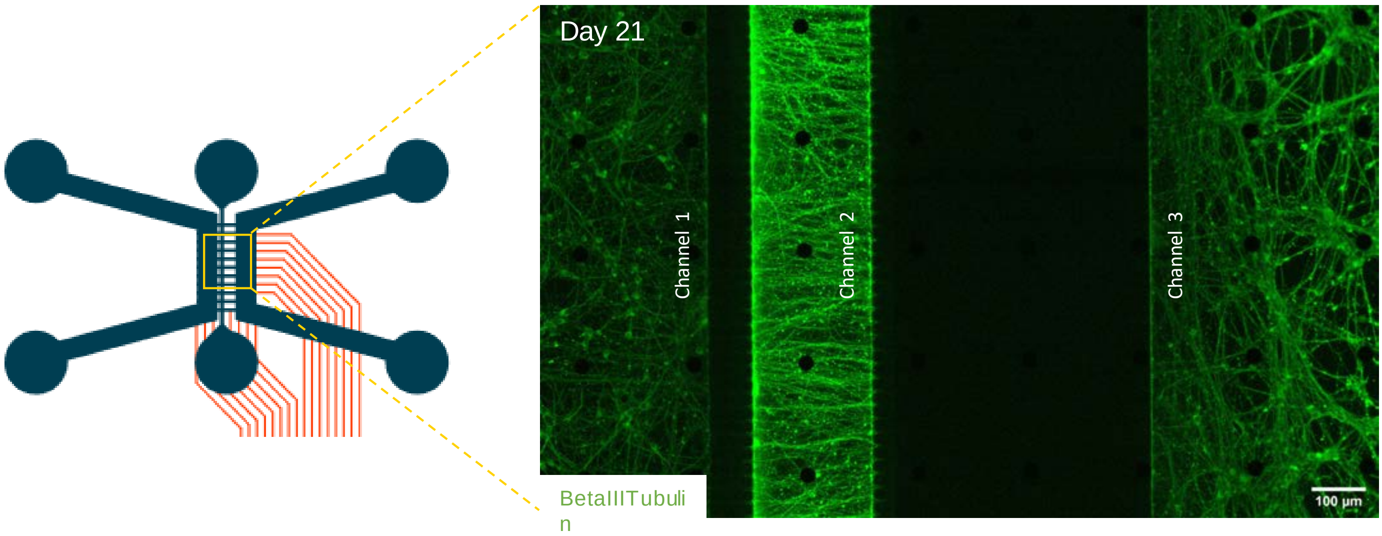

Figure S2: Immunofluorescence staining of human glutamatergic neurons stained with antibody anti-betaIIITubulin (green) in microfluidic device at day 21.

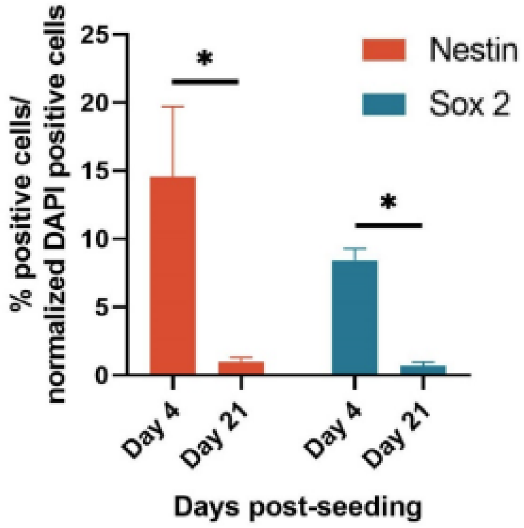

Figure S3: Graph showing the decrease of pluripotency markers in glutamatergic neurons maintained in microfluidic device with anti-Nestin and anti-Sox2 antibodies (* p-value <0.05, Student's t-test). The decrease in Nestin (red) and Sox 2 (blue) markers over time (between day 4 and 21 *in vitro*) indicates an optimal differentiation.

A

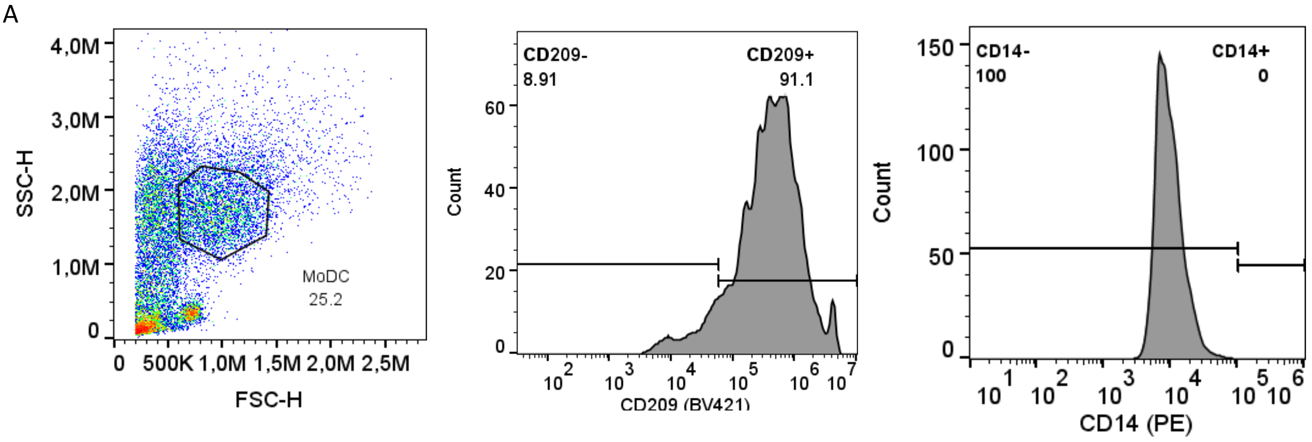

B

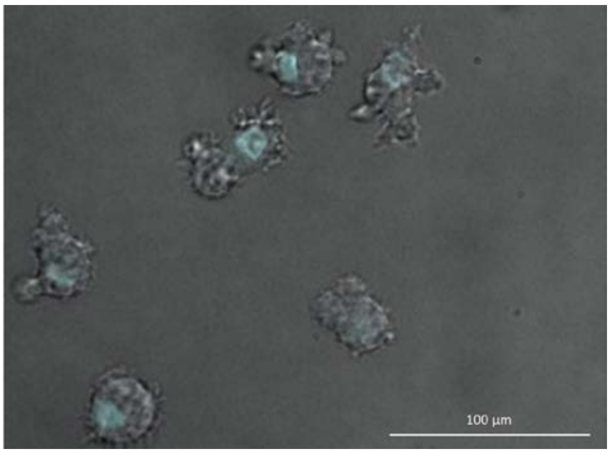

Figure S4: Monocyte-derived Dendritic cells (MoDCs) characterization.
A) Graph of Fluorescent Activated Cell Sorter (FACS) using CD209 antibody (increase) and CD14 antibody (decrease). The first graph shows the events regarding SSC and FSC on the total of a sample culture sample from CD14+ monocytes to dendritic cells presenting CD209+. Graph presenting the intensity of fluorescence signal on the gate of the first graph for CD209, attesting of a positivity, and for CD14, attesting of a negativity. This is confirming the phenotype of dendritic cells obtained by cytokine exposition differentiation.
B) Confocal picture of mature MoDCs X60 (Nikon Eclipse Ti2). This picture is showing the morphology of the dendritic cells obtained after differentiation by cytokine exposition of monocytes. Presence of pseudopods on the surface of cells is a guarantee of the differentiation to MoDC. The cells are marked by Hoescht. FSC: forward scatter, MoDC: monocyte-derived dendritic cell, SSC: side scatter.

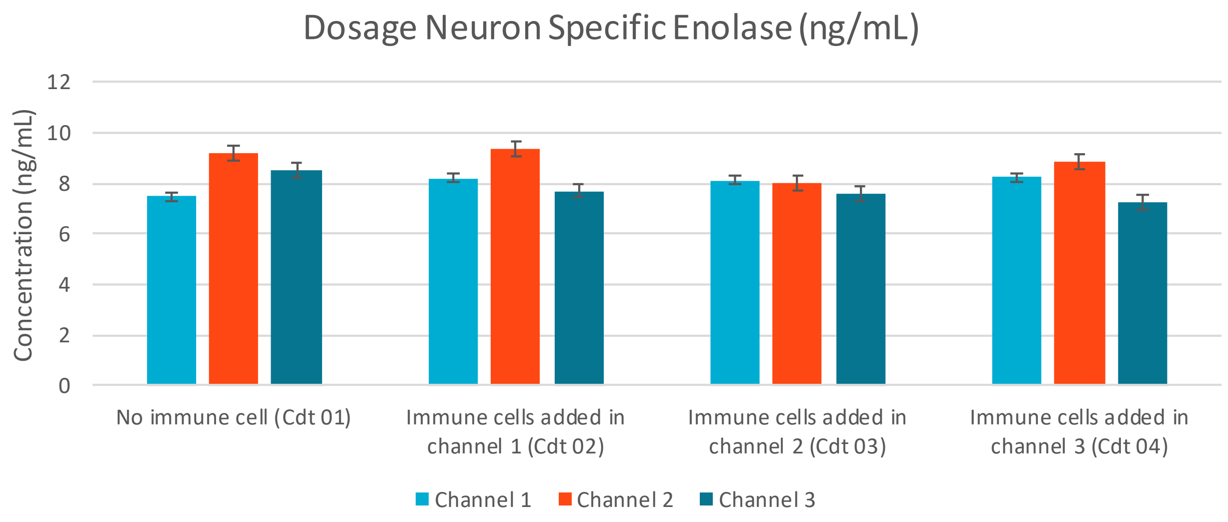

Figure S5: Graph of immunoassay of Neuron Specific Enolase in the supernatant of cell culture. The concentration of NSE (pg/ml) was quantified in the supernatant at day 21 of the human glutamatergic neurons culture, 4h after the addition of MoDCs. Quantification was performed in channels 1 (light blue), 2 (red), and 3 (dark blue). Results are presented as a function of conditions, detailed in Figure 2A. This marker is used as an indicator of neuronal death. No significative difference exists between the channel for this marker. Cdt: Condition, MoDCs: Monocyte-derived dendritic cells, NSE: Neuron Specific Enolase.

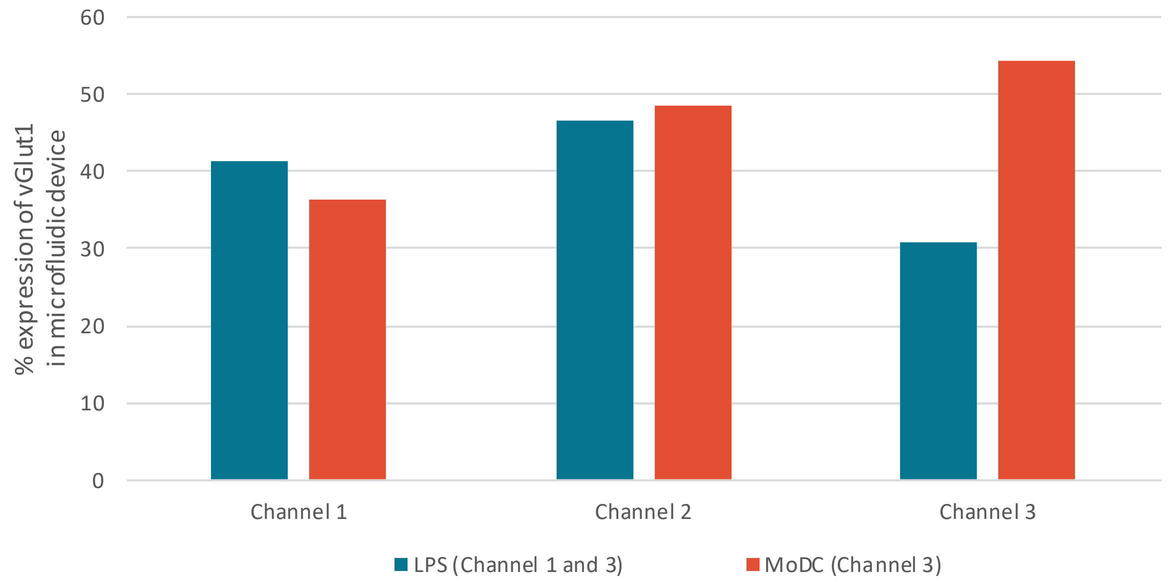

Figure S6: Graph of percentage of vGlut1 expression in human glutamatergic neurons according to the addition of MoDCs in microfluidic channel 3 (red) or LPS in channels 1 and 3 (blue). Quantification of vGlut1 is measured by fluorescence of immunostaining in the culture. vGlut 1 is a presynaptic protein responsible for glutamate transport into synaptic vesicles. LPS: Lipopolysaccharide, MoDCs: Monocyte-derived dendritic cells.

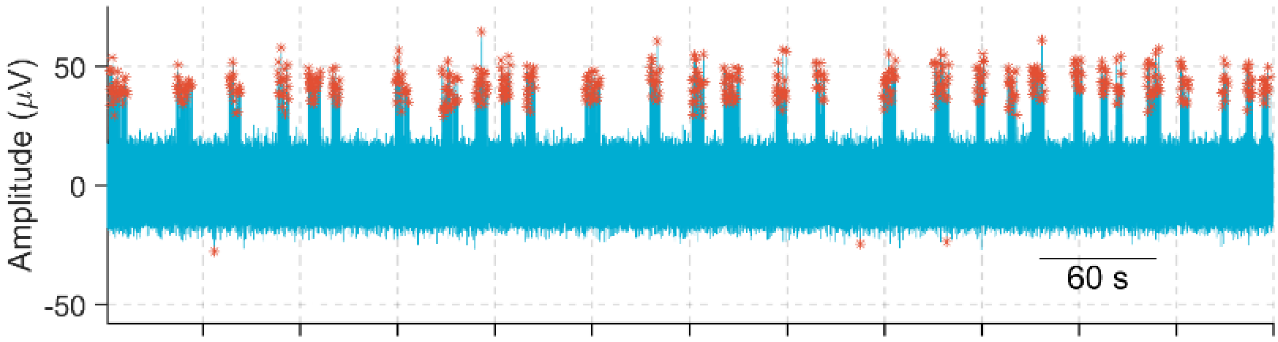

Figure S7: Plot of functional activity of human glutamatergic neurons seeded in asymmetric microfluidic device recorded at day 20 with MEA. The y-axis represents the amplitude of the spikes (marked with red dots) (µV) and the x-axis, the time, with a scale bar corresponding to 60 seconds (s). MEA: MicroElectrode Array.
